# Late Diagnosis of Familial Adenomatous Polyposis After Acute Lower Gastrointestinal Bleeding: A Case Report

**DOI:** 10.7759/cureus.76467

**Published:** 2024-12-27

**Authors:** Igor Logetto Caetité Gomes, Rômulo Silva Freire Junior, Luma Solidade Barreto, Luiz Carlos Cerqueira de Almeida Filho, Victor Alves Galvão

**Affiliations:** 1 Gastrointestinal Bleeding Center, Cleriston Andrade General Hospital, Feira de Santana, BRA

**Keywords:** adenomatous polyposis coli gene, colonic polyps, hereditary neoplastic syndromes, lower gastrointestinal hemorrhage, familial adenomatous polyposis

## Abstract

Familial adenomatous polyposis (FAP) is an autosomal dominant hereditary disease characterized by the progressive development of multiple adenomatous polyps along the colon. The majority of individuals develop colorectal cancer by the age of 40 within the evolutionary course of the disease. For this reason, screening family members is essential to enable identification, surveillance, and appropriate intervention. Colonoscopy is an essential examination in the follow-up of these patients. This case report aims to present an unusual case of a patient diagnosed with FAP after acute lower gastrointestinal bleeding at the age of 52. The recovery of the family history and the identification of multiple members with a mutation in the adenomatous polyposis coli (APC) gene supported the diagnosis.

## Introduction

Familial adenomatous polyposis (FAP) is characterized by the presence of multiple, more than 100, adenomatous polyps distributed throughout the colon and rectum. It is a rare autosomal dominant hereditary condition with an estimated prevalence of 2.29-3.2 cases per 100,000 individuals [1]. This condition is due to the germline mutation of the adenomatous polyposis coli (APC) gene, which can be either hereditary, which is the most common form, or a *de novo* mutation occurring during gametogenesis or embryogenesis, which is a less frequent form [2].

FAP has a genetic basis, and only one abnormal allele of the APC gene inherited from the parents is needed to express it. Another way of developing it is to be the first in the family to have the mutation and transmit it to your descendants. The mutation in the APC gene is responsible for the formation of adenomas, which progress sequentially to low-grade dysplasia, high-grade dysplasia, and, finally, colorectal adenocarcinoma [1].

In the initial phase, the disease can be asymptomatic and, as the adenomas develop and increase, rectal bleeding, anemia, abdominal discomfort, and weight loss can occur [3]. These polyps appear in the first two decades of life. Most of these patients will develop colorectal cancer by the age of 40 if they are not properly monitored and treated [4]. Other findings in the gastrointestinal tract include duodenal/gastric adenomas and gastric fundic gland polyps. Among the manifestations not related to the gastrointestinal tract, individuals with FAP are more likely to develop papillary thyroid carcinoma and its rare variant known as cribriform-morular thyroid carcinoma, desmoid tumors, and congenital hypertrophy of the retinal pigment epithelium [5,6].

## Case presentation

**Figure 1:**
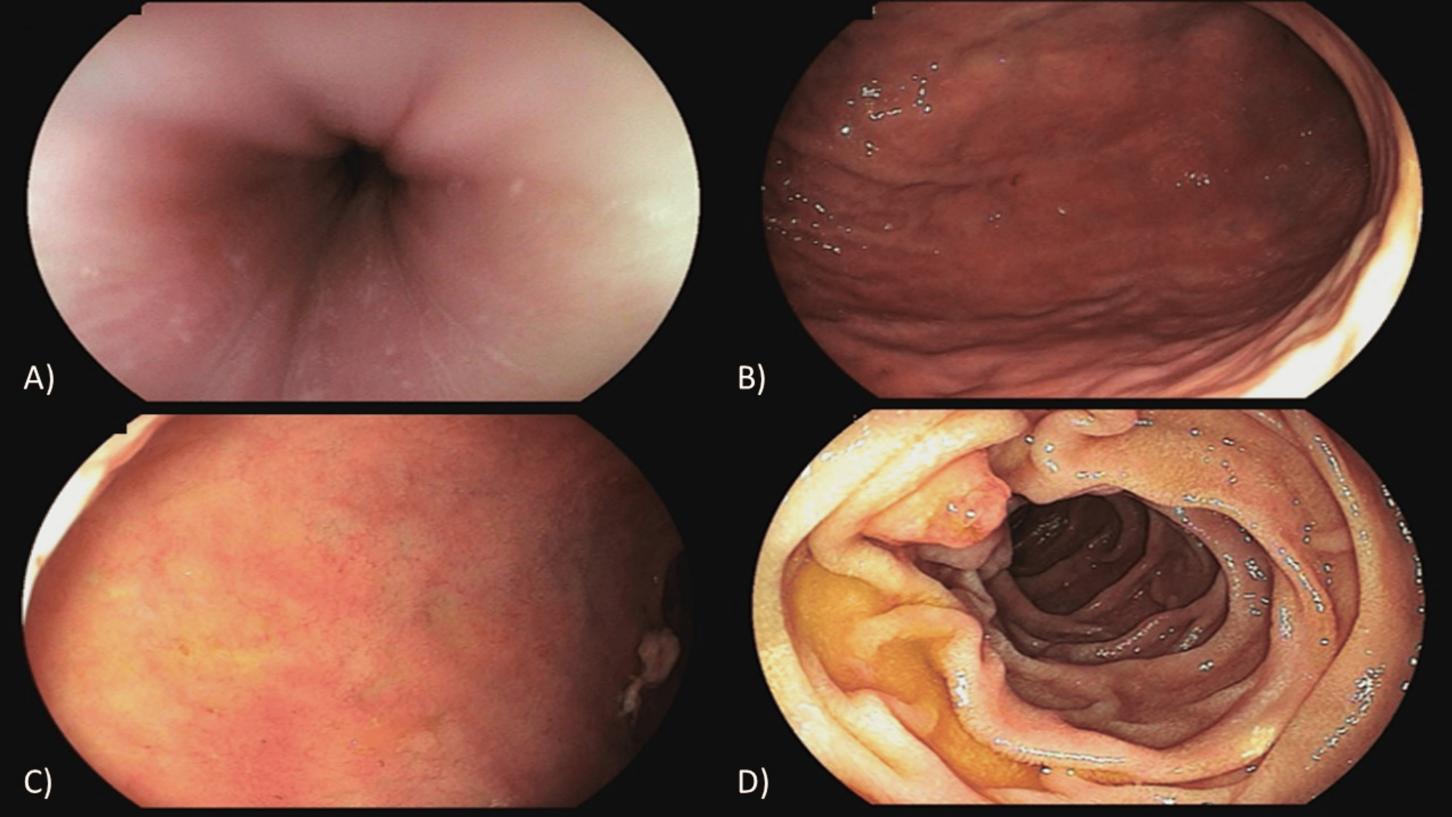
Upper digestive endoscopy: (A) esophagus without lesions; (B) no polyps observed in the stomach; (C) no lesions in the duodenal bulb; (D) normal second portion of the duodenum, with emphasis on the major papilla.

A 52-year-old man with systemic arterial hypertension and diabetes mellitus was admitted to hospital with acute lower gastrointestinal bleeding in the last 48 hours. The patient claimed that he had never presented any previous clinical manifestations related to the gastrointestinal tract and that he had come to the hospital because the bleeding had not stopped. During the rectal examination, the presence of blood and a mass in the rectum were identified, with no other relevant findings noted on physical examination. Laboratory tests revealed normochromic and normocytic anemia. He underwent an upper digestive endoscopy that revealed no relevant findings (Figure 1).

**Figure 2:**
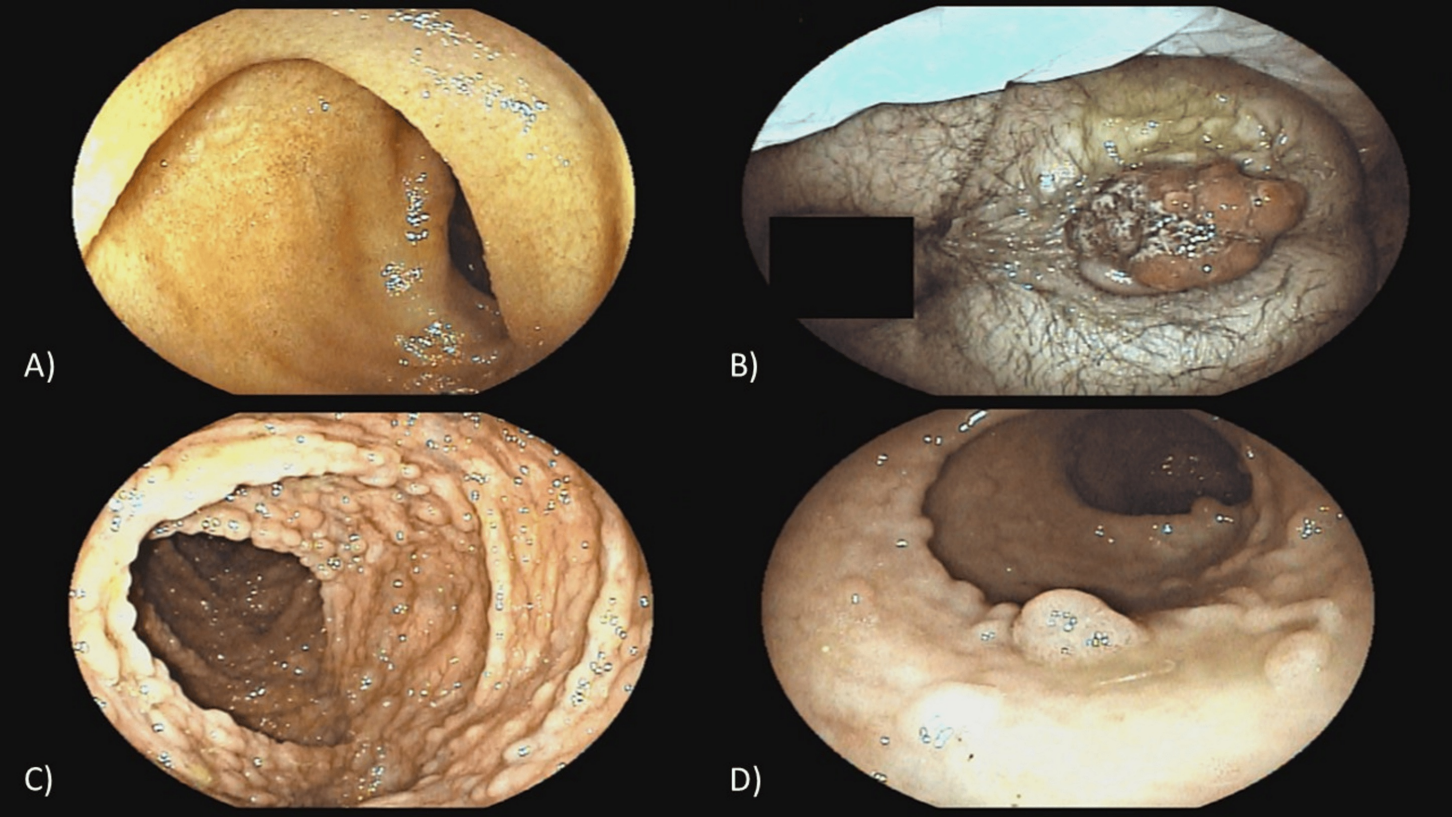
Colonoscopy: (A) no polyps in the intestinal ileum; (B) lesion in the rectum extending to the anal region; (C) multiple adenomatous polyps throughout the colon; (D) detailed view of one of the adenomatous polyps.

The patient underwent an antegrade preparation with 10% mannitol followed by a colonoscopy. During the procedure, a small amount of blood was identified in the rectum, along with the presence of numerous sessile polyps, more than 100, measuring between 5 and 13 mm. These polyps, displaying an adenomatous pattern, were diffusely distributed throughout all segments of the colon (Figure 2).

**Figure 3:**
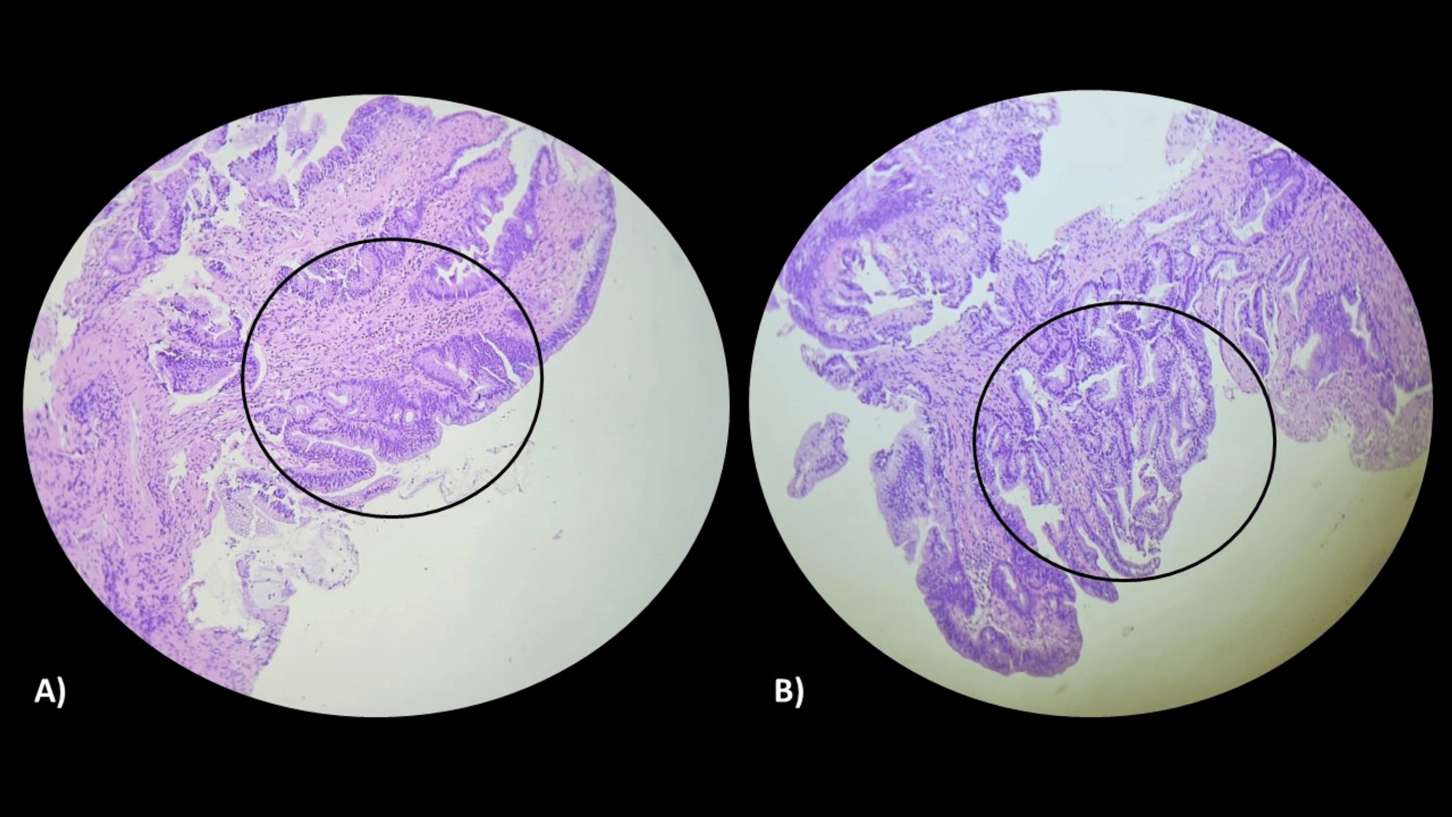
Histopathology: (A) microscopic examination showing an adenoma with high-grade dysplasia, highlighted in the circle (hematoxylin-eosin, 100×); (B) microscopic examination revealing an adenoma with low-grade dysplasia, highlighted in the circle (hematoxylin-eosin, 100×).

Polypectomies were performed by sampling multiple polyps with the cold snare. A larger, pedunculated, friable, bleeding polyp measuring 3 cm was also identified, located in the distal rectum, with exteriorization through the anal margin, which was biopsied. Histopathological analysis revealed that they were adenomas with low-grade dysplasia for the smaller polyps and high-grade dysplasia for the larger polyps (Figure 3).

The patient's family history was collected. A son, with whom the patient had not been in contact for many years, was identified and reported having a diagnosis of FAP. This diagnosis was established after persistent diarrhea led to a colonoscopy, during which numerous adenomatous polyps were identified and confirmed by histopathology. A molecular panel was subsequently performed, identifying the variant c.707T>G p.(Gln236Ter) in exon 7 of the APC gene in heterozygosis. This son had already undergone a total colectomy with ileorectal anastomosis and was being monitored every six to 12 months for the remaining rectal segment.

It was also identified that the patient's granddaughter and sister had received the same diagnosis after research revealed the same APC gene mutation profile, along with colonoscopies showing multiple adenomatous polyps. The patient was referred for multidisciplinary follow-up with a geneticist, gastroenterologist, digestive tract surgeon, psychologist, and oncologist. The ophthalmological examination did not reveal congenital hypertrophy of the retinal pigment epithelium, and the computed tomography scans showed no involvement of other organs.

In a joint decision, it was determined to perform a proctocolectomy with terminal ileostomy. Upon analysis of the entire specimen, numerous polyps of various morphologies and sizes were identified, including tubular, tubulovillous, and villous adenomatous types, associated with both low and high-grade dysplasia, but without evidence of adenocarcinoma. There were no signs of invasion, the surgical margins were free of dysplasia, and 28 free of disease were identified.

## Discussion

Colorectal cancer is a significant neoplasm and one of the most common cancers in both men and women in many Western countries. Even in developed countries, such as the United States, colorectal cancer remains the third leading cause of cancer-related death. Between 20% and 30% of these tumors have a familial origin, while approximately 70% to 80% are sporadic [7].

Patients with FAP are at high risk of developing colorectal cancer due to mutations in the APC gene. There are different variants of this mutation. The familial mutation pattern in the presented case was the c.707T>G p.(Gln236Ter) variant in exon 7. This variant creates a premature stop codon at amino acid 236, resulting in a truncated protein that increases susceptibility to cancer in these individuals [3]. 

Every adenoma presents some degree of dysplasia, characterized by cellular hyperchromatism, loss of polarity, hyperplasia, atypia, and other findings. From an evolutionary point of view, progressive changes in cellular architecture lead to the development of low-grade dysplasia, followed by high-grade dysplasia, and, finally, the formation of adenocarcinoma [8]. 

The ideal form of surveillance for colorectal cancer in these individuals is through a high-definition colonoscopy with white light. In general, surveillance should begin at 12 years of age and be conducted at one-year intervals, although there may be specific exceptions [9]. Adenomas are monitored through colonoscopy, and their number, size, histology, and location will support the decision on the type and timing of surgery. The patient's age must also be taken into account. The three main surgeries are subtotal or total colectomy with ileorectal or ileosigmoid anastomosis, restoration proctocolectomy (ileoanal anastomosis), and total proctocolectomy with end ileostomy [10].

The diagnosis at the age of 52 is an unusual finding present in this case report. When compared with the evidence in the scientific literature, it is possible to identify that almost all patients end up developing colorectal cancer by the age of 40 if they are not diagnosed and treated previously. Although, in rare cases, this can occur as late as 50-55 years of age [11].

A differential diagnosis to be considered in this case is the attenuated presentation of FAP. The attenuated form of FAP is characterized by fewer than 100 polyps, a greater distribution of polyps in the right colon that tends to spare the rectum, and a later diagnosis due to the slower progression of the disease [12,13]. Although the diagnosis in this case was made later, the patient had well over 100 polyps and diffuse involvement, including the rectum.

In the case of acute lower gastrointestinal bleeding in a 52-year-old patient, the initial hypotheses included diverticular disease, angiectasia, hemorrhoidal disease, or even sporadic colorectal cancer [14]. It was surprising to identify a hereditary condition, such as FAP, which is universally associated with the development of colorectal cancer at an earlier age.

Finally, the importance of a complete medical history should be emphasized. In the case presented, the detailed investigation made it possible to retrieve the family history even in the context of personal and social distancing from his first-degree relatives.

## Conclusions

As practically all patients develop colorectal cancer by the age of 40, the possibility of a diagnosis at the age of 52, as in the case presented, is rare. Therefore, when diagnosing the index case of FAP, it is essential to conduct an active and exhaustive search for first-degree relatives to provide early detection and the best prognosis for these family members. In the late stage of the disease, the patient may present with larger lesions in advanced phases that can manifest as acute lower gastrointestinal bleeding. Patients with FAP should be monitored by a multidisciplinary team, including a geneticist, gastroenterologist, surgeon, oncologist, psychologist, and nutritionist.
